# ST-Elevation Myocardial Infarction (STEMI) Due to Left Ventricular Thrombus Embolization: Implications of Multiagent Hormone Replacement Therapy

**DOI:** 10.7759/cureus.76951

**Published:** 2025-01-05

**Authors:** Hemraj Paudel, Hong Thoai Nguyen, Muhammad Sohaib Alvi, Aamir Abdullah

**Affiliations:** 1 Internal Medicine, Ascension Saint Joseph Hospital, Chicago, USA

**Keywords:** acute coronary syndrome (acs), anastrozole, cardioembolic, hormone replacement therapy (hrt), left ventricle thrombus, st-elevation myocardial infarction (stemi), tamoxifen therapy, testosterone

## Abstract

Acute coronary syndrome (ACS) primarily affects older individuals with established risk factors, but its occurrence in younger patients without traditional risk factors is rare and warrants attention. We present the case of a 41-year-old physically active male patient on long-term combination hormone replacement therapy (HRT) (testosterone, tamoxifen, and anastrozole) who developed ST-elevation myocardial infarction (STEMI) complicated by peri-infarction pericarditis (PIP). A diagnostic workup revealed a large thrombus occluding the left circumflex artery and a left ventricular (LV) thrombus. Despite attempts at thrombectomy, revascularization was unsuccessful, and the patient was managed with therapeutic anticoagulation, standard ACS pharmacotherapy, and cessation of hormone therapy. He was discharged on warfarin and continues regular follow-up at the anticoagulation clinic. His first follow-up visit to cardiology is yet to occur. This case highlights the potential cardiovascular risks associated with combined HRT, underscoring the need for clinicians and patients to be aware of its possible role in provoking thrombotic events and myocardial infarction in younger individuals.

## Introduction

Acute coronary syndrome (ACS), including ST-elevation myocardial infarction (STEMI), is typically associated with traditional cardiovascular risk factors such as smoking, hypertension, diabetes mellitus, dyslipidemia, and a family history of coronary artery disease (CAD). However, it can also occur in younger individuals with atypical risk profiles, particularly those undergoing hormone replacement therapy (HRT) [1,2]. We present the case of a 41-year-old male patient on long-term HRT for low-normal testosterone levels who presented to the emergency department with pleuritic chest pain and was diagnosed with STEMI. The patient had been using a combination of testosterone, tamoxifen, and anastrozole for 18 months, most likely initiated to manage low-normal testosterone and mitigate potential gynecomastia associated with testosterone therapy. This regimen led to complications, including a left ventricular (LV) thrombus leading to STEMI and peri-infarction pericarditis (PIP). This case is unique due to the use of a multiagent hormonal regimen without clear indications, the absence of traditional cardiovascular risk factors, and the significant thrombotic complications observed.

## Case presentation

A 41-year-old physically active male patient presented to the emergency department with a two-day history of central chest pain that began after a workout. The pain was pleuritic, more pronounced during inspiration, non-radiating, and worsened when lying supine but improved when sitting upright. He also reported associated symptoms of progressive fatigue, shortness of breath, palpitations, lightheadedness, diaphoresis, and nausea at the onset of the chest pain. However, these symptoms had resolved by the time of presentation. Initially attributing his symptoms to dehydration following exercise, he increased his fluid intake, which temporarily alleviated the pain. However, the pleuritic chest pain and severe fatigue persisted over the next two days. The patient sought evaluation at his primary care provider’s office for his scheduled testosterone injection, where he reported his symptoms. Following the injection, he was referred to the emergency department for further assessment of his chest pain. The patient described himself as highly active, regularly training in Brazilian jiu-jitsu training four times per week, each session lasting about two hours.

The patient reported that his serum testosterone levels were in the low-normal range (310-320 ng/dL) approximately 1.5 years prior. A review of his medical records revealed that he had been on intramuscular testosterone cypionate 125 mg weekly, oral tamoxifen 5 mg weekly, and oral anastrozole 0.25 mg weekly for the past 18 months. Baseline testosterone levels, basic labs, and coagulation profiles prior to therapy initiation were unavailable, limiting our understanding of his pretreatment hormonal and thrombotic risk. The patient denied any history of tobacco use, alcohol consumption, intravenous drug use, or illicit substance use. He had no known family history of CAD, sudden cardiac death, or hypercoagulable disorders. His past medical history was unremarkable, with no prior diagnosis of diabetes mellitus, hypertension, or dyslipidemia.

**Figure 1:**
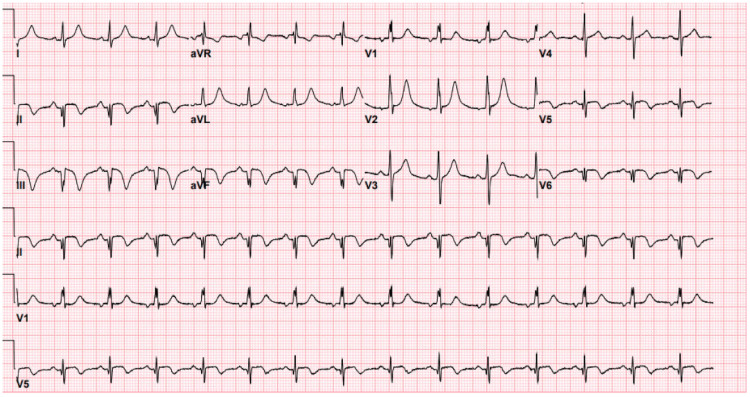
Twelve-lead electrocardiogram on admission

A 12-lead electrocardiogram (ECG) revealed left-axis deviation; ST-segment elevation; T-wave inversion in leads II, III, aVF, V5, and V6; and Q-waves in the inferior leads. These findings were consistent with inferolateral STEMI, implicating left circumflex (LCx) artery involvement due to the distribution of ischemia. Laboratory studies revealed a markedly elevated high-sensitivity troponin (hsT) level (>22,973 ng/mL), strongly indicating acute myocardial injury. His CBC and comprehensive metabolic panel (CMP) were unremarkable. The ECG findings are illustrated in Figure 1.

**Figure 2:**
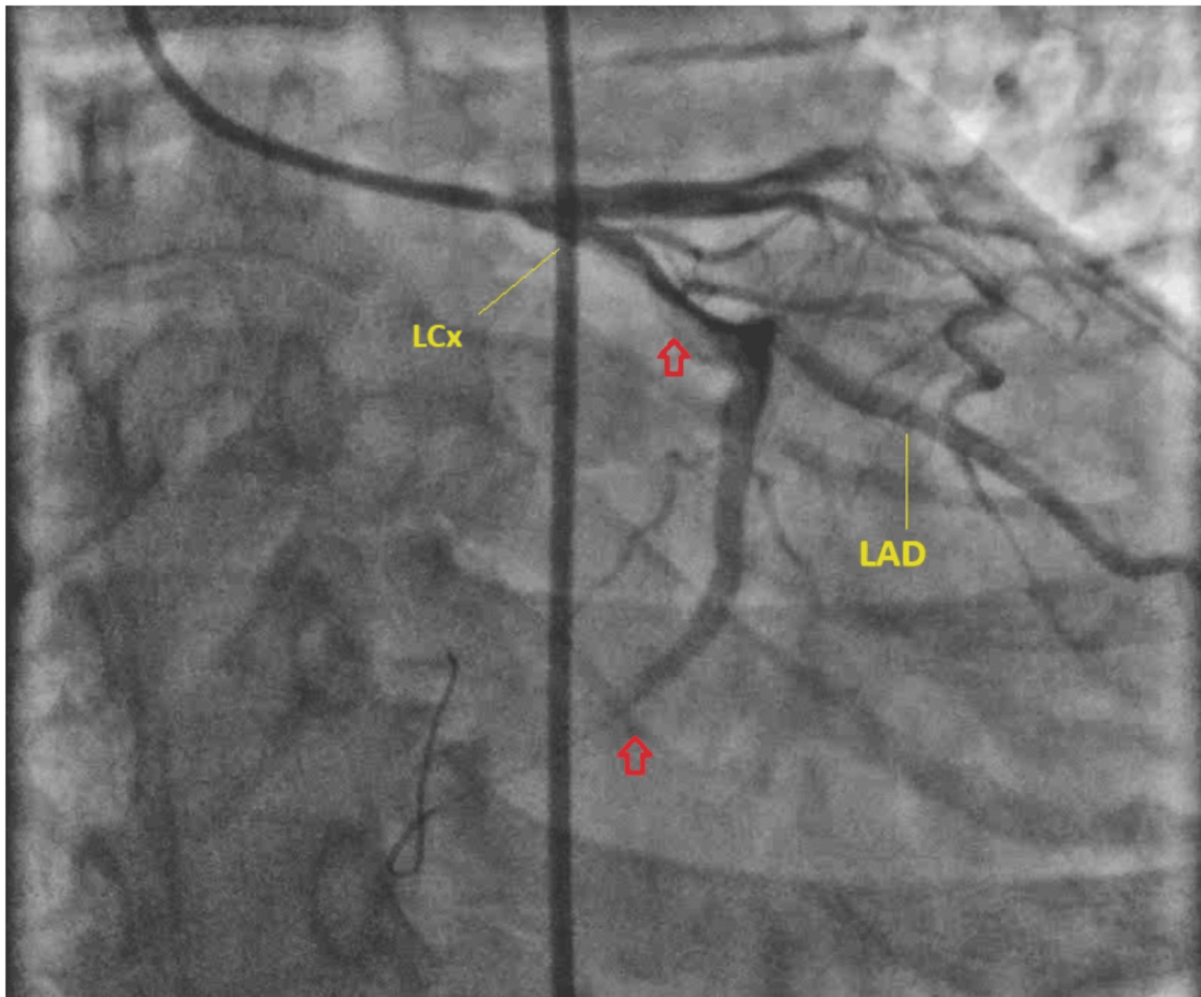
Coronary angiogram The upper red arrow points to the narrowing, indicating a large thrombus causing proximal LCx occlusion, while the lower red arrow indicates complete occlusion of the distal LCx. LCx: left circumflex artery, LAD: left anterior descending artery.

Left heart catheterization revealed a large thrombus in the proximal to the mid-left circumflex coronary artery and complete occlusion of the distal left circumflex artery in the atrioventricular groove segment. Due to the large thrombus, a thrombectomy was performed with the penumbra catheter. However, the minimal clot could be extracted as some degree of clot organization was suspected, likely due to delayed presentation and possible underlying hypercoagulability associated with HRT use. Minimal improvement in LCx flow was noted post-intervention. Thrombolysis in myocardial infarction (TIMI) 0 flow improved to TIMI 1 in the distal vessel, indicating a marginal restoration of perfusion. The clinical significance of TIMI flow grading in this context underscores the incomplete perfusion and potential for compromised outcomes. Given his late presentation, lack of active chest pain other than pleuritic, hemodynamic stability, pathologic Q-waves on ECG, and hsT of over 22,973, it was decided not to manipulate the thrombotic disease in the LCx further. This decision balanced the risks of additional intervention against the limited potential benefits due to the completed infarction. Left heart catheterization findings are illustrated in Figure 2.

**Figure 3:**
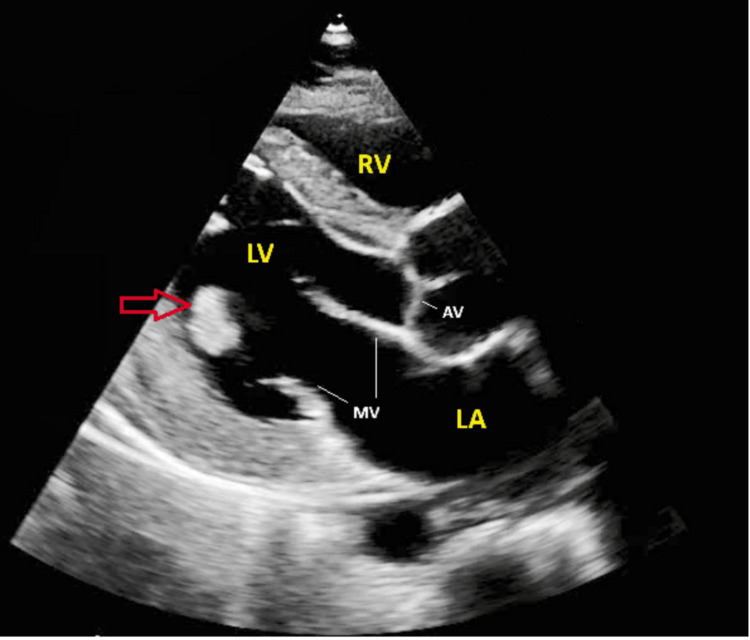
Transthoracic echocardiography The red arrow indicates a thrombus in the left ventricle (LV), attached to the papillary muscle and the posterior leaflet of the mitral valve. LV: left ventricle, RV: right ventricle, LA: left atrium, AV: aortic valve, MV: mitral valve.

Transthoracic echocardiography on the same day showed a normal-sized left ventricle with hypokinesis of the inferior basal segment (ejection fraction 55%). The atria were not dilated, there was a normal-sized right ventricle, and there were no significant valvular abnormalities or interatrial shunts. However, there was an echo density in the papillary muscle attached to the posterior leaflet with mild prolapse of the posterior leaflet, which was identified as a left ventricle thrombus. Hypercoagulable workup, including lupus anticoagulant, anticardiolipin antibodies, protein C activity, factor V Leiden mutation, and prothrombin gene mutation, was negative. A duplex ultrasound of the bilateral lower extremities showed no evidence of deep vein thrombosis, and plasma homocysteine levels were normal. His lipid panel and HB A1C were within normal limits. The cause of STEMI was suspected to be embolization of the LV thrombus. The patient was started on a therapeutic enoxaparin dose for five days to bridge to a therapeutic INR of 2.0-3.0 on warfarin and discharged on warfarin for three months. His discharge medications also included atorvastatin 80 mg daily, clopidogrel 75 mg daily, metoprolol 25 mg daily, and colchicine 0.6 mg daily (for PIP). He was advised to stop hormone replacement therapy and follow up with an endocrinologist for further workup after discharge. His first follow-up visit to the cardiology clinic is still pending, but he has been regularly attending the anticoagulation clinic to maintain a therapeutic INR while on warfarin. Transthoracic echocardiography findings are illustrated in Figure 3.

## Discussion

HRT has long been used to treat hypogonadism, support transgender healthcare, and assist certain cancer patients. Testosterone is the primary treatment for hypogonadism, but its side effects remain a concern. Its impact on the cardiovascular system, particularly in relation to CAD, has been the subject of significant debate. While some studies suggest neutral or potentially beneficial effects, case reports have linked testosterone use to acute myocardial infarction (AMI) [1,2]. The prothrombotic effects of testosterone, such as increasing hematocrit levels, platelet aggregation, and thromboxane A2 receptor density on platelets, are well-established and may contribute to cardiovascular events such as MI and stroke [3,4]. Studies also suggest that testosterone therapy in both transgender men and men with hypogonadism can adversely affect lipid metabolism, increasing low-density lipoprotein (LDL) cholesterol and decreasing high-density lipoprotein (HDL) cholesterol, further exacerbating cardiovascular risks [5-7]. These effects likely result from alterations in vascular tone and endothelial function, contributing to a prothrombotic state and potentially leading to LV thrombus formation.

Anastrozole and tamoxifen, commonly used in hormone-dependent cancers, have been associated with various cardiovascular effects, including arrhythmias, ischemic heart disease, and MI. The concurrent use of these medications may further elevate the risk of MI, especially in patients with other cardiovascular risk factors [8,9]. While tamoxifen has been shown to improve lipid profiles in some studies, it also decreases plasma HDL-C levels, which may increase the risk of atherosclerosis in certain populations, such as breast cancer patients [6,10]. The HER2 codon 655 G-allele has been linked to reduced HDL-C levels, further complicating the cardiovascular risk profile in patients undergoing tamoxifen therapy [10]. Tamoxifen, a derivative of diethylstilbestrol, has also been shown to increase the risk of cerebral venous thrombosis, highlighting the potential for thromboembolic events with hormonal therapy [11]. Similarly, while anastrozole has been shown to slightly increase the risk of MI and ischemic stroke, these risks have not been statistically significant [8,12]. In the ATAC trial, anastrozole was found to increase LDL cholesterol levels compared to tamoxifen, adding complexity to its cardiovascular risk profile [12]. Additionally, aromatase inhibitors like anastrozole have been associated with reduced endothelial function and potential vascular injury, contributing to an increased risk of cardiovascular disease [9,12].

Despite these findings, the adverse cardiovascular effects of HRT are not definitively established, and conflicting evidence underscores the need for further research to clarify these risks. Some studies suggest that testosterone therapy may have neutral or even beneficial effects on lipid profiles in certain populations [7]. However, the potential for thrombotic events, particularly in individuals with preexisting cardiovascular risk factors or those using high-dose or combined hormone therapies, remains a critical concern [1,2]. The Endocrine Society recommends testosterone therapy for adult males with hypogonadism to induce or maintain secondary sex characteristics and alleviate symptoms of testosterone deficiency [13]. For men over 65 years of age, therapy should be considered on a case-by-case basis after weighing potential risks and benefits, particularly in those with unequivocally low testosterone levels and associated symptoms such as low libido [13]. In patients with secondary hypogonadism who wish to preserve fertility, alternatives to testosterone therapy, such as human chorionic gonadotropin (hCG), selective estrogen receptor modulators (SERMs), and aromatase inhibitors (AIs), are available, although these options remain off-label and lack robust efficacy data [14]. Similar risks have been noted with androgen and anabolic steroid use, which can lead to extensive thrombus formation and subsequent embolism, further supporting the prothrombotic nature of testosterone therapy in this patient [3,15].

Our patient presented with pleuritic chest pain indicative of a completed infarction leading to PIP. The patient’s atypical chest pain, the presence of a large LV thrombus on the day of presentation, and the absence of atherosclerotic plaques on coronary angiography suggest that the lesion was cardioembolic, likely stemming from the thrombotic effects of HRT [15]. The patient had been receiving weekly testosterone cypionate injections for 18 months, alongside tamoxifen and anastrozole. This dosage exceeded the recommended levels, and the concurrent use of two off-label treatments likely amplified the prothrombotic effects. However, there is a lack of sufficient studies on the combined use of testosterone, anastrozole, and tamoxifen in young, physically active men without traditional cardiovascular risk factors. The simultaneous use of these three agents could potentially create a "perfect storm" for LV thrombus formation in our patient. Although the literature describes limited prothrombotic effects of single or dual agents [1,6,10], the combined impact remains unexplored and represents a significant knowledge gap. This area warrants further research to better understand the associated risks. However, the development of LV thrombus post-MI involves endothelial injury, blood stasis, and hypercoagulability, which are all factors that contribute to thrombus formation and embolization [15]. The cessation of hormone therapy is a critical intervention in this case, yet balancing the benefits of HRT with its potential cardiovascular risks remains a challenge, particularly in patients requiring treatment for conditions like hypogonadism or certain cancers. Individualized risk assessment, including consideration of cardiovascular profiles and lifestyle factors, is essential to optimize therapy while minimizing harm. Future research is needed to better understand the long-term cardiovascular effects of HRT, especially in young, active patients without traditional risk factors, to guide safer and more effective treatment strategies.

## Conclusions

This case highlights the importance of recognizing unconventional etiologies of AMI in young patients, particularly those undergoing HRT. The concurrent use of multiple hormonal agents, as demonstrated in this case, underscores the potential for compounding thrombotic risks, leading to severe cardiovascular complications such as left ventricular thrombus and embolic myocardial infarction. These findings emphasize the necessity of judicious prescribing practices, thorough risk assessment, and vigilant monitoring in patients receiving HRT. Further research is imperative to elucidate the mechanisms and safety profiles of multiagent hormonal therapies, thereby informing clinical guidelines and mitigating adverse outcomes. Educating patients about the potential risks and ensuring prompt evaluation of cardiovascular symptoms are critical for improving outcomes in this population. Current guidelines for testosterone replacement therapy recommend monitoring hematocrit, baseline and follow-up lipid panels, and PSA levels but do not address whether monitoring coagulation profiles or echocardiographic evaluations improves outcomes. This highlights a gap in the monitoring protocols for patients on multi-agent HRT, particularly those at risk for cardiovascular events. Further research is needed to establish comprehensive monitoring guidelines to mitigate potential thrombotic and embolic risks associated with such therapies.
